# Design and Experimental Evaluation of a Shoulder Assistive Exoskeleton for Insulator Replacement

**DOI:** 10.3390/s26082313

**Published:** 2026-04-09

**Authors:** Haoyuan Chen, Jia Yao, Ming Li, Hongwei Hu, Zhan Yang, Siyu Tu, Yalun Liu, Zimeng Wang, Zhao Guo

**Affiliations:** 1 State Grid Hubei Electric Power Co., Ltd., Extra High Voltage Company, Wuhan 430050, China; chenhy58@hb.sgcc.com.cn (H.C.); lim260@hb.sgcc.com.cn (M.L.); huhw@hb.sgcc.com.cn (H.H.); yangz11@hb.sgcc.com.cn (Z.Y.); a766698@163.com (S.T.); 2 School of Power and Mechanical Engineering, Wuhan University, Wuhan 430072, China; 2025202080006@whu.edu.cn (J.Y.); 2025102080024@whu.edu.cn (Y.L.); 2022302191187@whu.edu.cn (Z.W.); 3 School of Robotics, Wuhan University, Wuhan 430072, China

**Keywords:** upper-limb assistive exoskeleton, insulator replacement, dynamic modeling, sEMG signal analysis

## Abstract

Aiming to reduce muscle fatigue and prevent occupational injuries caused by prolonged lifting in insulator replacement operations, this study presents the design of an upper-limb exoskeleton. Firstly, this study performs kinematic analysis and phase segmentation of the lifting motion in the insulator replacement operation. Based on the analysis, in terms of mechanical structure, the proposed upper-limb exoskeleton adopts a unilateral three-degree-of-freedom shoulder mechanism that biomimics the human glenohumeral joint, which reduces the misalignment between the exoskeleton and the human body. Meanwhile, a waist–back support structure is integrated into the exoskeleton to realize a more reasonable torque transmission path. In terms of the control strategy, based on the operation’s phase segmentation and dynamic modeling of the human upper limb, this study develops a neural network-based assistive control algorithm for insulator replacement operations, enabling the exoskeleton to provide phase-specific torque output. Experimental results demonstrate that, under a simulated insulator replacement operation with a 20 kg load, the exoskeleton significantly reduces the subject’s sEMG activity of the biceps brachii and triceps brachii, effectively alleviating muscle fatigue.

## 1. Introduction

High-voltage transmission line insulator replacement is a critical routine maintenance operation in power transmission systems [1,2]. Due to the large mass of the insulators, maintenance personnel are required to climb towers tens of meters high and perform prolonged high-intensity lifting operations in strong electromagnetic fields. Such highly repetitive and intensive actions impose excessive loads on the shoulder, back, and arm muscle groups, which easily lead to acute fatigue and occupational diseases such as lumbar muscle strain [3,4,5]. This not only threatens the health of workers but also affects their working efficiency. As a type of human–machine collaborative intelligent equipment, exoskeleton robots can provide external torque assistance to specific joints of the user [6], significantly reducing the user’s metabolic consumption and muscle activation levels during operation execution [7,8], and directly enhancing the user’s specific motor capabilities [9,10]. They have been widely applied in multiple fields such as industry [11], military [12], and medical rehabilitation [13]. Although upper-limb assistive exoskeletons for actions such as material handling have emerged in the industrial field [14], there is still no research on upper-limb assistive exoskeletons specifically targeting insulator replacement scenarios. Therefore, the development of an upper-limb assistive exoskeleton dedicated to assisting insulator replacement operations and alleviating user muscle fatigue has become an urgent problem to be addressed in both the power industry and the robotics field.

According to whether a power source is used, existing upper-limb assistive exoskeletons can be mainly classified into two categories: passive and active types. Passive upper-limb assistive exoskeletons usually utilize energy storage elements such as springs and elastic soft materials to store and release energy, thereby achieving assistive effects [15,16,17]. However, such passive exoskeletons generally share common drawbacks: the magnitude and stroke of the assistance they provide are fixed and cannot be dynamically adjusted according to actual loads and motion postures, and the maximum assistive torque is relatively low, making it difficult to meet the demand for continuous lifting of insulators in high-voltage transmission line maintenance [18,19].

In contrast, active upper-limb assistive exoskeletons use motors [20], hydraulic systems [21], or pneumatic components [22] as power sources, enabling controllable and adjustable active assistance with higher assistive output [23,24], and are therefore more suitable for lifting insulators with large mass. Accordingly, this paper mainly focuses on active exoskeletons. In the field of industrial manufacturing, Panasonic of Japan developed the AWN03, which is motor-driven and can reduce workers’ lower-back stress by 15 kg, with an endurance of 8 h [25]. Vatan et al. from the University of Salford developed a wearable cable-driven shoulder exoskeleton that employs a Bowden cable transmission mechanism inspired by biological tendons. The device is capable of reducing user load, and experiments conducted with healthy subjects showed position errors of less than 5% [26]. Guo et al. from Harbin Institute of Technology developed a cable-driven upper-limb exoskeleton suit that employs a pneumatic sensing glove to detect hand grasping states and uses motors to drive the cables, thereby effectively assisting upper-limb actions such as pulling and lifting. Experimental results demonstrate that this exoskeleton can significantly reduce the activation levels of major muscles [27].

However, for the demands of insulator replacement on high-voltage transmission lines, existing active upper-limb assistive exoskeletons still face several limitations [28]. In terms of mechanical design, most active upper-limb assistive exoskeletons typically model the shoulder joint as a simple three-degree-of-freedom spherical joint, without accounting for the displacement of the shoulder’s instantaneous center of rotation during motion. This misalignment between the exoskeleton and the human body reduces wearing comfort. In addition, many active upper-limb assistive exoskeletons retain an excessive number of actuators for the sake of generality, resulting in bulky systems that fail to meet the lightweight requirements of high-altitude operations. Moreover, most existing active upper-limb assistive exoskeletons lack efficient lumbar–back support structures and reasonable torque transmission pathways, making it difficult to redistribute the load borne by the upper limbs to the torso. This not only significantly reduces assistance efficiency but also increases the risk of secondary fatigue in the user’s lower back. Regarding control strategies, assistive algorithms specifically designed for insulator replacement operations on high-voltage transmission lines are still very limited. Existing control methods are insufficient for handling the dynamically changing user postures characteristic of this operation, making it difficult to achieve appropriately adjusted assistive torque output.

To address the aforementioned issues, this paper designs an upper-limb assistive exoskeleton tailored to the requirements of power maintenance personnel performing insulator replacement operations. The main contributions of this study are as follows: First, a kinematic analysis of the assistive motions involved in high-voltage transmission line insulator replacement operations is conducted, and the operation is segmented into distinct phases. Based on this analysis, in terms of mechanical structure, a lightweight upper-limb exoskeleton with good wearability is designed. Specifically, a unilateral three-degree-of-freedom shoulder mechanism biomimicking the human glenohumeral joint is proposed, enabling the exoskeleton motion to better conform to the human scapulohumeral rhythm and thereby mitigate misalignment issues during human–exoskeleton coupled motion. In addition, a parallel movable dual-link support structure is integrated at the waist and back to transfer part of the load to the user’s back, forming a rational torque transmission path and substantially reducing the mechanical load on the user’s arms. In terms of the control strategy, based on the phase segmentation and dynamic modeling of the human upper limb of the insulator replacement operation, this study develops a convolutional neural network-based control strategy for assisting insulator replacement operations. Human joint angles are measured in real time to determine the current motion phase, and phase-specific assistive torques are applied accordingly. Finally, experimental results demonstrate that, under a simulated insulator replacement operation with a 20 kg load, wearing the proposed exoskeleton significantly reduces sEMG activity of the biceps brachii and triceps brachii, effectively alleviating muscle fatigue.

The rest of this article is organized as follows: Section 2 presents the design and kinematic modeling of the upper-limb exoskeleton, dynamic modeling of the human upper limb, and control strategy of the exoskeleton, followed by the description of the experimental protocol. Section 3 describes the experimental data analysis, and Section 4 discusses the results and concludes this article.

## 2. Materials and Methods

### 2.1. Design of Exoskeleton for Insulator Replacement Operation

#### 2.1.1. Kinematic Analysis of Insulator Replacement Operation

In high-voltage transmission line insulator replacement operations, the core objective of an upper-limb assistive exoskeleton is to provide the user with the required joint torques at the upper limbs to lift insulators, thereby reducing arm effort. To ensure wearing comfort and safety during the lifting of heavy objects, it is necessary to systematically analyze the kinematic characteristics of the upper-limb joints throughout the operation, ensuring that the mechanical structure of the designed exoskeleton does not interfere with the natural motions of the upper limbs.

**Figure 1:**
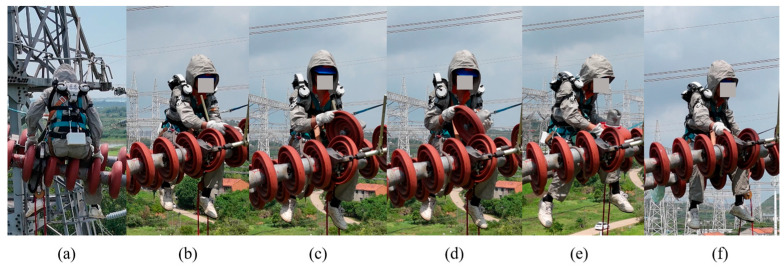
Phase segmentation of insulator replacement operation: (**a**) preparation phase; (**b**) reaching phase; (**c**) lifting phase; (**d**) holding phase; (**e**) lowering phase; (**f**) returning phase.

Based on the movements of power grid maintenance personnel during insulator replacement [29], the lifting operation is divided into five phases, as shown in Figure 1:

(a) Preparation phase: The operator sits upright with arms positioned at the sides of the body.

(b) Reaching phase: The operator leans forward and extends the arms to grasp the insulator.

(c) Lifting phase: After grasping the insulator, the operator uses the arms and torso to raise the insulator from a position directly in front of the thighs to the chest level.

(d) Holding phase: The operator maintains the insulator at chest level for a short period to inspect it.

(e) Lowering phase: The operator carefully lowers the insulator back to a position near the feet.

(f) Returning phase: After lowering the insulator, the operator returns the arms from the forward position to the sides of the body, restoring the initial upright posture.

**Figure 2:**
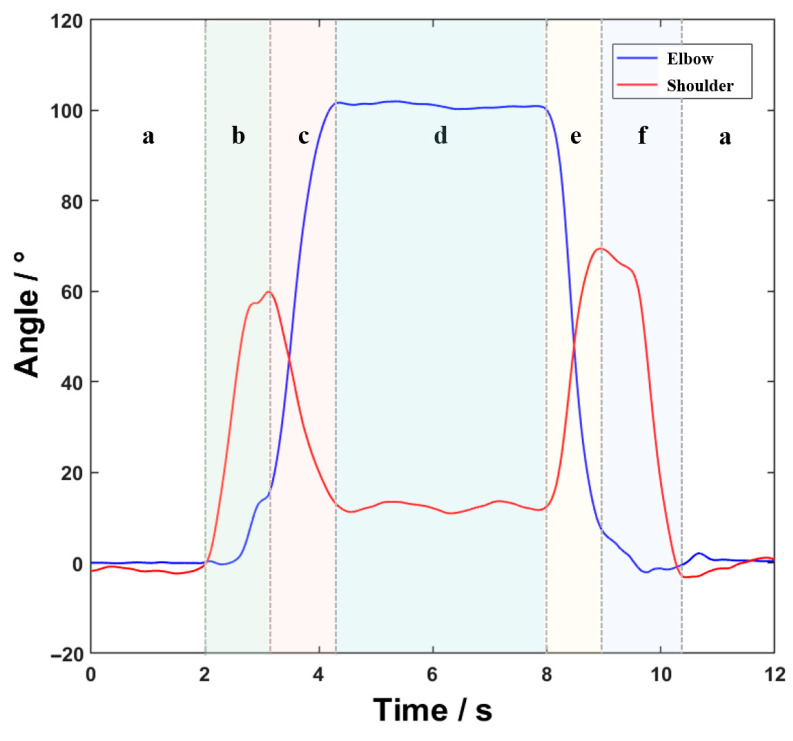
The joint angle trajectories during the operation process: (**a**) preparation phase; (**b**) reaching phase; (**c**) lifting phase; (**d**) holding phase; (**e**) lowering phase; (**f**) returning phase.

We collected IMU data on the flexion and extension angles of the shoulder and elbow joints in the sagittal plane during lifting tasks from 8 healthy adult participants (all with experience in electrical work), and then normalized and averaged the data. The resulting joint angle trajectories during the operation process are shown in Figure 2, and the corresponding numerical values are presented in Table 1.

#### 2.1.2. Structural Design

Based on the kinematic analysis and phase segmentation of the insulator replacement operation on high-voltage transmission lines, the proposed active upper-limb assistive exoskeleton is configured with three degrees of freedom (DOFs) on each side: shoulder flexion/extension, abduction/adduction, and internal/external rotation. Among them, shoulder flexion/extension and abduction/adduction, which account for the majority of motion during the insulator replacement operation, are designed as active DOFs. In contrast, the internal/external rotation DOF, which contributes less to the overall motion, is designed as a passive DOF that can be self-adjusted by the user. The DOF configuration of the exoskeleton and the corresponding joint ranges of motion are listed in Table 2.

During human shoulder adduction/abduction, the rotation center of the glenohumeral joint shifts with the degree of abduction. To better accommodate this variation in the rotational center and to follow the scapulohumeral rhythm, the exoskeleton developed in this study adopts a biomimetic design of the human glenohumeral joint by placing the motor for the adduction/abduction degree of freedom on the back. When the user performs movements while wearing the exoskeleton, the rotation center of the exoskeleton’s shoulder joint correspondingly shifts, thereby reducing misalignment between the exoskeleton and the human body. In addition, the proposed exoskeleton adopts a parallel movable dual-link support structure at the waist, which transfers part of the load to the user’s back and reduces the load borne by the arms, thus enhancing the user’s load-carrying capability. The exoskeleton adopts carbon fiber and resin materials for its main structure and 7075 aluminum alloy for critical load-bearing components, with each actuator weighing approximately 550 g. As a result, the total mass of the exoskeleton is only 4.5 kg, and a single side can provide an assistive torque of up to 50 N·m, achieving a high power density of 22.2 N·m/kg.

**Figure 3:**
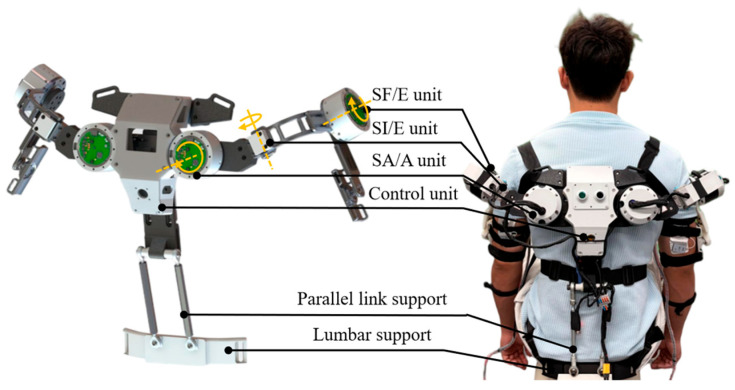
Overall structure of the exoskeleton.

In terms of wearing configuration, the back of the upper-limb assistive exoskeleton is fitted to the user’s back via shoulder straps, the user’s arms are tightly secured to the exoskeleton arms using flexible straps, and the waist is fixed to the human body through a waist belt. To accommodate inter-individual variations in body dimensions, the exoskeleton incorporates adjustable mechanisms at the waist–back segment and the upper arm, allowing for customized length adjustment. Detailed adjustment parameters are shown in Table 3. The overall mechanical structure and the wearing configuration are illustrated in Figure 3.

#### 2.1.3. Hardware Design

**Figure 4:**
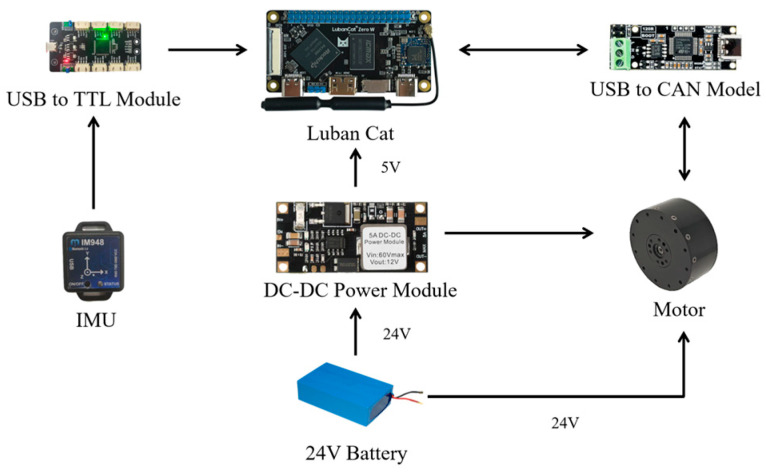
Hardware design of the exoskeleton.

The hardware design of the exoskeleton is shown in Figure 4. A 24 V lithium battery is used as the power supply of the exoskeleton. A LubanCat is adopted as the main controller, and a voltage conversion module is employed to provide a 5 V power supply to the LubanCat. The actuators are Quanzhibo motors (Wuxi Quanzhibo Technology Co., Ltd., Wuxi, China), and communication between the motors and the LubanCat is realized through a USB-to-CAN module. To obtain accurate human motion data and achieve more precise motor torque control, IMUs are deployed on the back of the exoskeleton as well as on the upper arm, forearm, and back of the operator to acquire the joint angles and angular velocities of the human shoulder and elbow joints. A USB-to-TTL module is used to enable communication between the IMUs and the LubanCat.

### 2.2. Kinematic Modeling of the Exoskeleton

**Figure 5:**
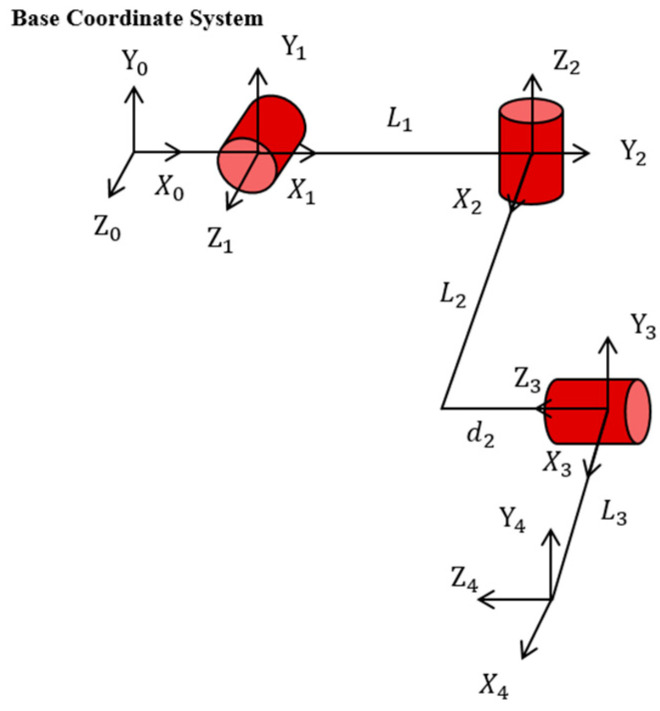
MD–H model of the exoskeleton. $O_{0}$, $O_{1}$, $O_{2}$, $O_{3}$ and $O_{4}$ denote the base coordinate frame and the coordinate frames of the shoulder adduction/abduction, internal/external rotation, and flexion/extension joints, respectively.

To facilitate intuitive understanding, the Modified Denavit–Hartenberg (MD–H) method is adopted to establish the coordinate frames of the upper-limb exoskeleton. For clarity of presentation, only the unilateral upper-limb exoskeleton was modeled, with the MD-H parameters of the exoskeleton listed in Table 4. A base coordinate frame $O_{0}$ is first established. Then, coordinate frames $O_{1}$, $O_{2}$, $O_{3}$ and $O_{4}$ are successively assigned to the shoulder adduction/abduction, internal/external rotation, and flexion/extension joints and the robot end, respectively. The origin of the base frame $O_{0}$ coincides with that of the shoulder adduction/abduction frame $O_{1}$. For clarity in the illustration, the $O_{0}$ coordinate frame is shifted leftward in the drawing, and the simplified MD–H model is shown in Figure 5.

Once the MD–H parameters are determined, the homogeneous transformation matrix Tii−1 of each coordinate frame relative to the previous frame can be obtained using the spatial coordinate transformation formula [30]:

(1) Tii−1=Rot(Xi−1,αi−1)Trans(Xi−1,ai−1)Rot(Zi,θi)Trans(Zi,di)

The transformation matrix between adjacent links in space is given by

(2) Tii−1=cθi−sθi0ai−1sθicαi−1cθicαi−1−sαi−1−disαi−1sθisαi−1cθisαi−1cαi−1dicαi−10001

where cθ and sθ denote cosθ and sinθ, respectively.

By substituting the parameters into Equation (2), the transformation matrices between adjacent joints Tii−1, for i=1:4, can be calculated.

(3) T10=Rot(Z1,θ1)=cθ1−sθ100sθ1cθ10000100001

(4) T21=Rot(X1,α1)Trans(X1,L1)Rot(Z2,θ2)=cθ2−sθ20L10010−sθ2−cθ2000001

(5) T32=Rot(X2,α2)Trans(X2,L2)Rot(Z3,θ3)Trans(Z3,d2)=cθ3−sθ30L200−1d2sθ3cθ3000001

(6) T43=Trans(X3,L3)=100L3010000100001

The relationship between the end-effector coordinate frame of the designed upper-limb exoskeleton and the base coordinate frame is given by T40 as follows:

(7) T40=T10T21T32T43=nxoxaxpxnyoyaypynzozazpz0001

By substituting the numerical values, the following is obtained:

(8) T40=c1c2c3−s1s3−c1c2s3−s1c3c1s2c1(c2L2−s2d2+L1+c2c3L3)−s1s3L3s1c2c3+c1s3−s1c2s3+c1c3s1s2s1(c2L2−s2d2+L1+c2c3L3)+c1s3L3−s2c3s2s3c2−s2(L2+c3L3)−c2d20001

where $c_{1}$, $c_{2}$ and $c_{3}$ denote $\cos\theta_{1}$, $\cos\theta_{2}$ and $\cos\theta_{3}$; $s_{1}$, $s_{2}$ and $s_{3}$ denote $\sin\theta_{1}$, $\sin\theta_{2}$ and $\sin\theta_{3}$.

**Figure 6:**
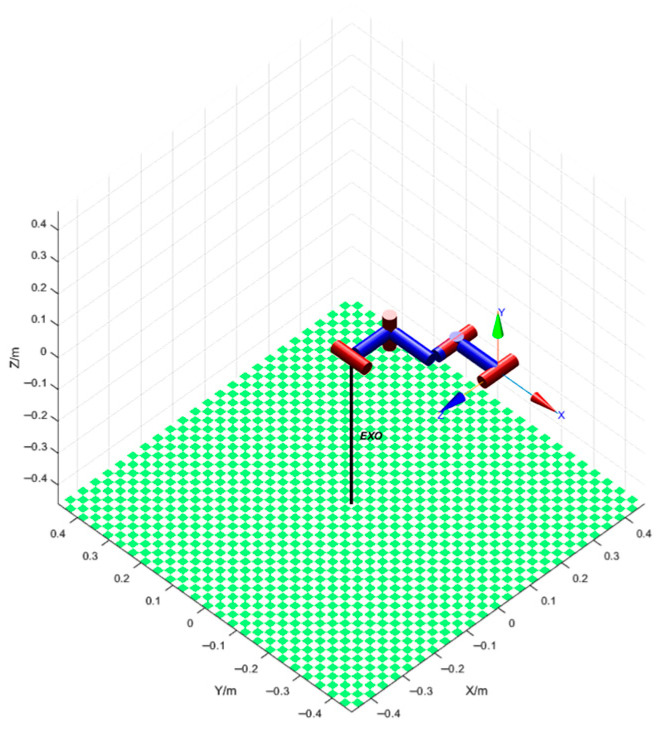
Model of the upper-limb exoskeleton.

**Figure 7:**
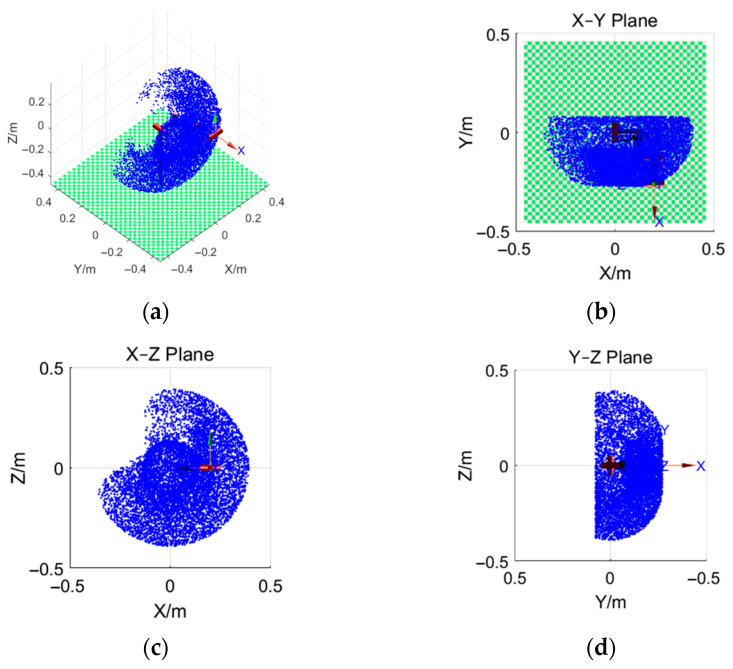
Workspace point cloud of the exoskeleton. (**a**) represents the 3D point cloud of the workspace; (**b**) represents the XY-plane point cloud of the workspace; (**c**) represents the XZ-plane point cloud of the workspace; (**d**) represents the YZ-plane point cloud of the workspace.

The designed upper-limb exoskeleton was modeled in MATLAB 2022 to analyze the workspace, as shown in Figure 6. Subsequently, the Monte Carlo random sampling method was employed to visualize the robot workspace [31]. Let $L_{1}=0.12\;m$, $L_{2}=0.13\;m$, $L_{3}=0.13\;m$, and $d_{2}=0.08\;m$; MATLAB was used to randomly generate 10,000 samples of $\theta_{1}$, $\theta_{2}$, and $\theta_{3}$, where $-150{}^{\circ}\leq \theta_{1}\leq 90{}^{\circ}$, $-180{}^{\circ}\leq \theta_{2}\leq 0{}^{\circ}$, and $-150{}^{\circ}\leq \theta_{3}\leq 90{}^{\circ}$. These values were substituted into Equation (8) to obtain 10,000 sample points, which collectively reflect the reachable workspace of the exoskeleton robot end-effector, as shown in Figure 7a–d. In the figures, the blue region represents the reachable workspace of the unilateral exoskeleton end. It can be seen that the designed exoskeleton robot is basically consistent with the actual upper-limb activity space of the human body during the insulator-lifting operation.

### 2.3. Dynamic Modeling of Human Upper Limb

Given the relatively small mass of the upper-arm link of the exoskeleton, a simplified dynamic model was established by considering only the human upper limb during the lifting task for shoulder joint torque control of the exoskeleton.

The system dynamics can be formulated as follows:

(9) $$M(q)\ddot{q}+C(q,\dot{q})\dot{q}+G(q)=\tau$$

**Figure 8:**
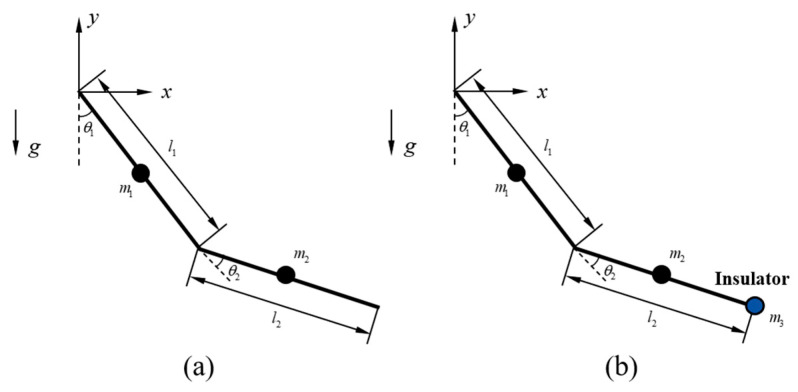
Kinematic and dynamic model of the human upper limb. (**a**) Unloaded dynamic model; (**b**) loaded dynamic model.

Since the upper-limb motion during the lifting task primarily occurs in the sagittal plane, a simplified model was adopted. The unilateral human upper limb was modeled as a two-link mechanism with only flexion–extension degrees of freedom, and each link was assumed to be a homogeneous rigid body, as shown in Figure 8.

In this model, $l_{1}$ and $l_{2}$ denote the lengths of the upper arm and forearm, respectively; $m_{1}$ and $m_{2}$ denote their corresponding masses; and $\theta_{1}$ and $\theta_{2}$ represent the shoulder and elbow joint angles. The insulator was modeled as a point mass attached to the distal end of the forearm, with mass $m_{3}$.

Without the insulator load, the parameters of the human–exoskeleton upper-limb dynamic model are defined as follows:

(10) $$M(\theta )=\left[\begin{array}{cc} \left(\frac{1}{3}m_{1}+m_{2}\right)l_{1}^{2}+\frac{1}{3}m_{2}l_{2}^{2}+m_{2}l_{1}l_{2}\cos\theta_{2} & \frac{1}{3}m_{2}l_{2}^{2}+\frac{1}{2}m_{2}l_{1}l_{2}\cos\theta_{2}\\ \frac{1}{3}m_{2}l_{2}^{2}+\frac{1}{2}m_{2}l_{1}l_{2}\cos\theta_{2} & \frac{1}{3}m_{2}l_{2}^{2} \end{array}\right]$$

(11) $$C(\theta ,\dot{\theta })=\left[\begin{array}{cc} 0 & -\frac{1}{2}m_{2}l_{1}l_{2}(2{\dot{\theta }}_{1}+{\dot{\theta }}_{2})\sin\theta_{2}\\ \frac{1}{2}m_{2}l_{1}l_{2}{\dot{\theta }}_{1}\sin\theta_{2} & 0 \end{array}\right]$$

(12) $$G(\theta )=\left[\begin{array}{c} \left(\frac{1}{2}m_{1}+m_{2}\right)gl_{1}\sin\theta_{1}+\frac{1}{2}m_{2}gl_{2}\sin(\theta_{1}+\theta_{2})\\ \frac{1}{2}m_{2}gl_{2}\sin(\theta_{1}+\theta_{2}) \end{array}\right]$$

With the insulator load, the parameters of the human–exoskeleton upper-limb dynamic model are defined as follows:

(13) $$M(\theta )=\left[\begin{array}{cc} M_{11} & M_{12}\\ M_{21} & M_{22} \end{array}\right]$$

(14) $$C(\theta ,\dot{\theta })=\left[\begin{array}{cc} 0 & -\frac{1}{2}(m_{2}+2m_{3})l_{1}l_{2}(2{\dot{\theta }}_{1}+{\dot{\theta }}_{2})\sin\theta_{2}\\ \frac{1}{2}(m_{2}+2m_{3})l_{1}l_{2}{\dot{\theta }}_{1}\sin\theta_{2} & 0 \end{array}\right]$$

(15) $$G(\theta )=\left[\begin{array}{c} \left(\frac{1}{2}m_{1}+m_{2}+m_{3}\right)gl_{1}\sin\theta_{1}+\left(\frac{1}{2}m_{2}+2m_{3}\right)gl_{2}\sin(\theta_{1}+\theta_{2})\\ \frac{1}{2}\left(m_{2}+2m_{3}\right)gl_{2}\sin(\theta_{1}+\theta_{2}) \end{array}\right]$$

where $M_{ij}$ are given by

(16) $$\left\{\begin{array}{l} M_{11}=\left(\frac{1}{3}m_{1}+m_{2}+m_{3}\right)l_{1}^{2}+\left(\frac{1}{3}m_{2}+m_{3}\right)l_{2}^{2}+(m_{2}+2m_{3})l_{1}l_{2}\cos\theta_{2}\\ M_{12}=\left(\frac{1}{3}m_{2}+m_{3}\right)l_{2}^{2}+\frac{1}{2}(m_{2}+2m_{3})l_{1}l_{2}\cos\theta_{2}\\ M_{21}=\left(\frac{1}{3}m_{2}+m_{3}\right)l_{2}^{2}+\frac{1}{2}(m_{2}+2m_{3})l_{1}l_{2}\cos\theta_{2}\\ M_{22}=\left(\frac{1}{3}m_{2}+m_{3}\right)l_{2}^{2} \end{array}\right.$$

### 2.4. Control System

In this study, we designed a control strategy based on convolutional neural networks (CNNs) and a finite state machine (FSM), specifically for assisting insulator replacement operations. This control strategy can determine the phases of the insulator replacement operation in real time and adjust the exoskeleton torque accordingly. A convolutional neural network model is employed for phase prediction. The model architecture consists of convolutional layers, a fully connected layer, a convolution transpose layer, and a softmax layer. The input data include human shoulder and elbow joint angles and angular velocity signals, while the output corresponds to the user’s current phase in the insulator replacement operation. The first step of the control process involves continuously collecting the operator’s shoulder and elbow joint angles and angular velocities along with the corresponding insulator replacement operation phases. These data are then fed into the CNN for offline training, and the trained model is saved in LubanCat for online use.

**Figure 9:**
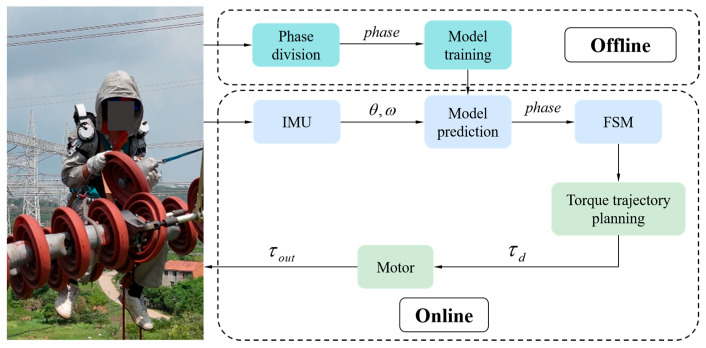
Flowchart of the exoskeleton control strategy.

During the real-time control of the lifting process in insulator replacement, IMUs continuously acquire the human shoulder and elbow joint angles and angular velocity signals, which are input into the offline-trained neural network model for phase determination. The determined phase information is then fed into the finite state machine, thereby enabling exoskeleton state transitions and phase-specific assistive torque. In phases (b) and (f), the exoskeleton joint torque is computed using the unloaded dynamic model; in phases (c), (d) and (e), it is computed using the loaded model; and in phase (a), the exoskeleton does not provide active torque. The workflow of this control strategy is shown in Figure 9.

### 2.5. Experiments

An experiment was conducted in which a subject wore the upper-limb assistive exoskeleton to perform a simulated insulator replacement lifting operation, in order to evaluate the assistive effect of the exoskeleton.

**Figure 10:**
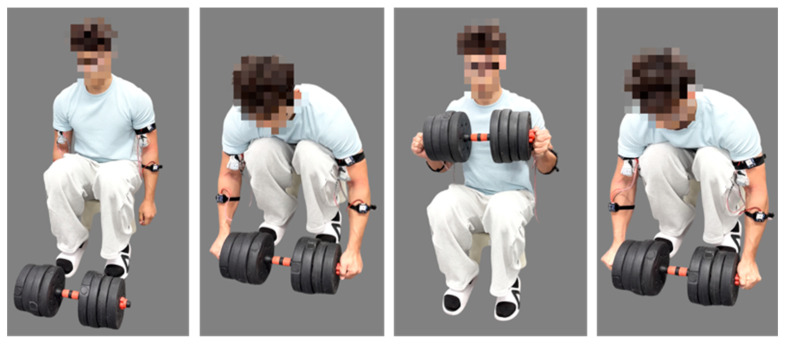
Experiment without the exoskeleton.

**Figure 11:**
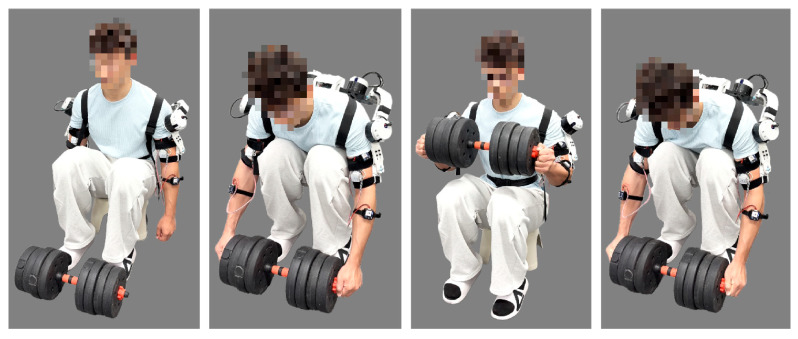
Experiment with the exoskeleton.

Depending on the application scenarios of insulators, their weight ranges from 15 to 20 kg. Therefore, a load of 20 kg was selected to simulate the insulator replacement scenario, in order to verify the assistive effect of the exoskeleton. In the experiment, the subject performed lifting trials with and without the exoskeleton while completing a simulated insulator replacement lifting operation with a 20 kg load. Each trial consisted of five repetitions of lifting the load from the ground to the same position in front of the chest, holding it for a period, and then lowering it back to the same position on the ground, while attempting to maintain a consistent range of motion in each repetition. The repetitions were performed discontinuously. Five healthy subjects were recruited to participate in the experiments; the subject information is summarized in Table 5. The experimental setup is shown in Figure 10 and Figure 11.

To evaluate the assistive performance of the exoskeleton, we collected the angular changes of IMUs of the shoulder joints and sEMG signals of the biceps brachii and triceps brachii while the subject performed lifting motions with and without the assistive exoskeleton. The sEMG signals were acquired using the Zhiyunwei wireless EMG system (Shanghai Jingchang Industrial Co., Ltd., Shanghai, China) at a sampling frequency of 1000 Hz. The measurement principle of sEMG is to attach electrodes to the skin surface to capture electrical signals generated during muscle activity. The signal amplitude varies positively with the extent of muscle movement, allowing sEMG signals to accurately reflect muscle activation levels. Prior to attaching the EMG sensors to the subject’s skin, the attachment areas were cleaned with alcohol to reduce impedance during signal acquisition. When the shoulder and elbow joints perform different movements, the corresponding muscle sEMG signals fluctuate accordingly, thereby reflecting the activation levels of the respective muscles.

To better illustrate the experimental results, the overall root mean square (RMS) values of sEMG signals during a single lifting trial by a subject were used to reflect their variations during the experiment. The RMS effectively represents the amplitude of the sEMG signals, reflects the real-time intensity of muscle activation, and suppresses noise to a certain extent.

The RMS is calculated as follows:

(17) $$RMS=\sqrt{\frac{\sum_{i=1}^{N}{\left|x_{\left(i\right)}\right|}^{2}}{N}}$$

Here, N represents the number of samples in each window.

In order to calculate the RMS values of the sEMG, a band-pass filter was applied to a set of raw sEMG data. RMS values were extracted using a window length of 20 with a sliding step of 10, and the extracted data were subsequently processed with a Gaussian filter.

The overall root mean square (RMS), integrated electromyography (IEMG), and maximum absolute value (MAXABS) of the sEMG signals were calculated for each of the five repetitions in each trial for every participant, and the mean values of these parameters were then obtained. Subsequently, the relative change rates in the mean values of these parameters with and without the exoskeleton were computed for each participant. The change rate with and without exoskeleton was calculated as follows:

(18) $$(M_{\mathrm{EXO}}-M_{\mathrm{NEXO}})/M_{\mathrm{NEXO}}\times 100\%.$$

Finally, the mean and standard deviation of the change rates of these parameters, as well as the between-group *p* values were calculated across all participants to evaluate the effect of the exoskeleton on upper-limb muscle activation. Specifically, IEMG represents the cumulative muscle activity over a specified time period and reflects the overall level of muscle activation. MAXABS represents the peak instantaneous muscle activation and indicates the maximum contraction intensity of the muscle.

## 3. Results

**Figure 12:**
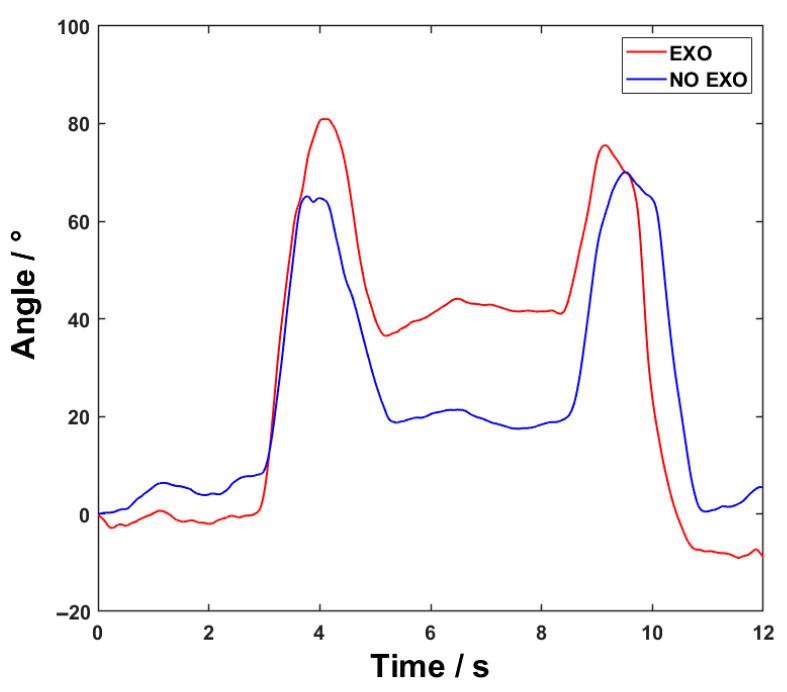
The shoulder joint angle variations of a subject with and without the exoskeleton.

The shoulder joint angle variations of a subject during the experiment are shown in Figure 12. The results indicate that, when wearing the exoskeleton, the subject exhibits a significantly greater shoulder joint range of motion compared to the condition without the exoskeleton. The increase can be attributed to the assistive torque provided by the exoskeleton throughout the lifting task. This assistive torque partially reduces the load borne by the subjects. Moreover, under the mechanical constraints of the exoskeleton, the subjects are able to achieve a greater motion amplitude while remaining within a safe range. All subjects reported no discomfort caused by the mechanical structure during use.

**Figure 13:**
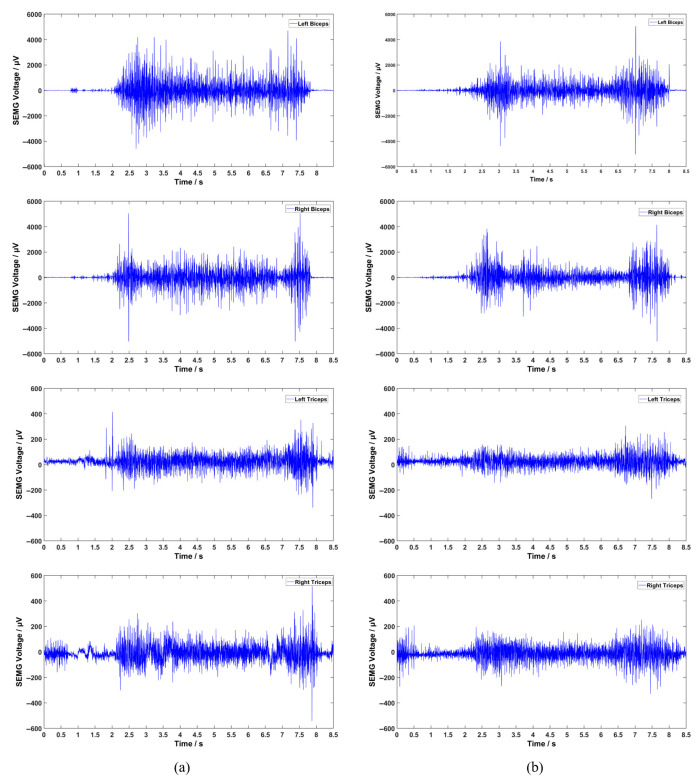
Raw experiment data. (**a**) represents data without the exoskeleton; (**b**) represents data with the exoskeleton.

**Figure 14:**
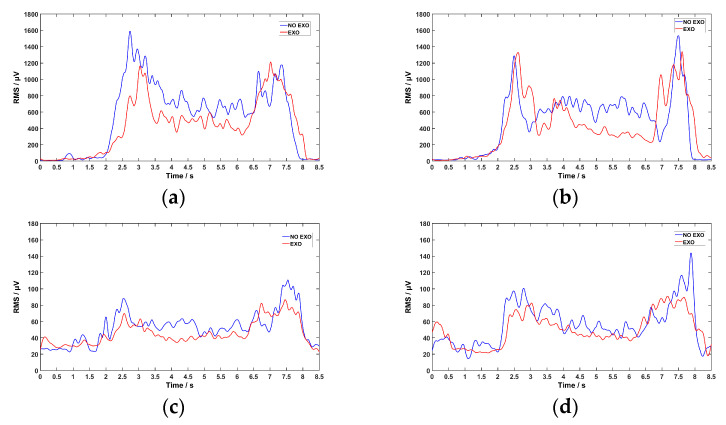
Comparison of RMS data for four muscles. (**a**) represents RMS comparison of left biceps brachii; (**b**) represents RMS comparison of right biceps brachii; (**c**) represents RMS comparison of left triceps brachii; (**d**) represents RMS comparison of right triceps brachii.

A set of raw sEMG data from the experiment is shown in Figure 13. To better illustrate the experimental results, the RMS values of sEMG signals during a single lifting trial by a subject were used to reflect their variations during the experiment. The results of the experimental analysis are shown in Figure 14.

As illustrated, compared to performing a single lifting motion without the exoskeleton, wearing the exoskeleton led to a decrease in RMS values for the left biceps brachii, left triceps brachii, right biceps brachii, and right triceps brachii. This indicates that the energy expenditure of these muscles was reduced.

To eliminate potential errors from a single trial and better illustrate the experimental effect, every subject performed a total of five lifting trials. Detailed experimental data are shown in Table 6.

Based on the table, the following conclusions can be drawn: Under exoskeleton assistance, the RMS, IEMG, and MAXABS values of multiple muscles in the participants all exhibited significant reductions. These results indicate that when the subject performed weighted lifting while wearing the exoskeleton, the muscles were relatively relaxed, with a lower average activation level.

## 4. Discussion and Conclusions

This study addresses the demands of high-voltage transmission line insulator replacement operations by proposing a high-performance upper-limb assistive exoskeleton system specifically designed for this scenario. Based on kinematic analysis and phase segmentation of the assistive lifting motions during insulator replacement operations, the exoskeleton features a humanoid-inspired and lightweight mechanical structure. It adopts a unilateral three-degree-of-freedom shoulder mechanism that biomimics the human glenohumeral joint, allowing the exoskeleton’s motion to better follow the natural shoulder–arm kinematics. A parallel movable dual-link support is implemented in the waist–back region, significantly reducing the load on the user’s arms. In addition, based on the phase segmentation of the insulator replacement task and dynamic modeling of the human upper limb, this study develops a neural network-based assistive control algorithm for insulator replacement operations. Experimental results demonstrate that the exoskeleton provides effective assistance during the lifting motions of simulated insulator replacement operations, reducing the RMS, IEMG and MAXABS of sEMG signals in the involved muscles, thereby addressing the lack of exoskeleton robots specifically designed for insulator replacement in the power maintenance field.

For future work, structural improvements will focus on optimizing the exoskeleton’s DOF configuration and adding active assistance at the elbow joint to better suit insulator replacement operations. In terms of control, more adaptive and generalizable strategies will be developed to further enhance the exoskeleton’s performance. Furthermore, on-site experiments of actual insulator replacement will be conducted to validate the effectiveness of the exoskeleton.

## Figures and Tables

**Table 1:** Joint angle variations during different phases.

Phases	Shoulder Joint Angle (Flexion/Extension)	Elbow Joint Angle (Flexion/Extension)
(a) Preparation phase	~0°	~0°
(b) Reaching phase	Increase to ~60°	Increase to ~15°
(c) Lifting phase	Reduce to ~15°	Increase to ~100°
(d) Holding phase	The same as (c)	The same as (c)
(e) Lowering phase	Increase to ~60°	Reduce to ~15°
(f) Returning phase	Reduce to ~0°	Reduce to ~0°

**Table 2:** DOF configuration and joint range of motion of the exoskeleton.

Joint	DOF	Plane of Motion	Range of Motion
Shoulder joint	flexion/extension	sagittal plane	0° to 60°/0° to 180°
	abduction/adduction	coronal plane	0° to 60°/0° to 180°
	internal/external rotation	transverse plane	0° to 90°/0° to 90°

**Table 3:** Adjustable range of the exoskeleton.

Components	Adjustment Range (mm)
back length	475–495
shoulder thickness	110–130
shoulder width	490–510
arm length	110–150

**Table 4:** The MD–H parameters of the unilateral exoskeleton.

i	$\alpha_{i-1}$	$a_{i-1}$	$d_{i}$	$\theta_{i}$ (Initial Value)	$\mathrm{Range}\;\mathrm{of}\;\theta_{i}$
1	0°	0	0	$\theta_{1}(0{}^{\circ})$	−150° to 90°
2	−90°	$L_{1}$	0	$\theta_{2}(-90{}^{\circ})$	−180° to 0°
3	90°	$L_{2}$	$-d_{2}$	$\theta_{3}(0{}^{\circ})$	−150° to 90°
4	0°	$L_{3}$	0	0°(0°)	0°

**Table 5:** The subject information.

Subject	Sex	Age (years)	Height (cm)	Weight (kg)
S1	M	21	175	72.5
S2	M	24	168	70.0
S3	M	25	176	75.5
S4	M	25	180	75.0
S5	M	27	175	73.0

**Table 6:** Effects of exoskeleton wear on sEMG signals during the lifting task.

	Parameters	RMS			IEMG			MAXAbs		
Muscle										
		**Mean**	**STD**	***p***	**Mean**	**STD**	***p***	**Mean**	**STD**	***p***
Left biceps brachii		−33.26%	7.40%	<0.01	−26.91%	9.48%	<0.01	−33.02%	4.41%	<0.01
Right biceps brachii		−20.40%	10.31%	0.025	−16.4%	9.2%	0.037	−21.51%	12.41%	0.031
Left triceps brachii		−10.87%	5.97%	0.036	−9.54%	3.39%	0.048	−15.73%	6.71%	0.022
Right triceps brachii		−13.33%	6.38%	0.015	−7.87%	4.54%	0.040	−20.69%	11.77%	0.048

In the table, Mean denotes the mean change rate, STD denotes the standard deviation of the change rate, and *p* denotes the *p* value for the between-group difference in muscle activation.

## Data Availability

The data presented in this study are available on request from the corresponding author.
